# FTY720 Treatment in the Convalescence Period Improves Functional Recovery and Reduces Reactive Astrogliosis in Photothrombotic Stroke

**DOI:** 10.1371/journal.pone.0070124

**Published:** 2013-07-31

**Authors:** Robert Brunkhorst, Nathalie Kanaan, Alexander Koch, Nerea Ferreirós, Ana Mirceska, Pia Zeiner, Michel Mittelbronn, Amin Derouiche, Helmuth Steinmetz, Christian Foerch, Josef Pfeilschifter, Waltraud Pfeilschifter

**Affiliations:** 1 Department of Neurology, Goethe University Hospital, Frankfurt am Main, Germany; 2 Department of General Pharmacology and Toxicology, Goethe University Hospital, Frankfurt am Main, Germany; 3 Department of Clinical Pharmacology, Goethe University Hospital, Frankfurt am Main, Germany; 4 Department of Neuropathology, Goethe University Hospital, Frankfurt am Main, Germany; 5 Department of Anatomy II, Goethe University Hospital, Frankfurt am Main, Germany; Julius-Maximilians-Universität Würzburg, Germany

## Abstract

**Background:**

The Sphingosine-1-phosphate (S1P) signaling pathway is known to influence pathophysiological processes within the brain and the synthetic S1P analog FTY720 has been shown to provide neuroprotection in experimental models of acute stroke. However, the effects of a manipulation of S1P signaling at later time points after experimental stroke have not yet been investigated. We examined whether a relatively late initiation of a FTY720 treatment has a positive effect on long-term neurological outcome with a focus on reactive astrogliosis, synapses and neurotrophic factors.

**Methods:**

We induced photothrombotic stroke (PT) in adult C57BL/6J mice and allowed them to recover for three days. Starting on post-stroke day 3, mice were treated with FTY720 (1 mg/kg b.i.d.) for 5 days. Behavioral outcome was observed until day 31 after photothrombosis and periinfarct cortical tissue was analyzed using tandem mass-spectrometry, TaqMan®analysis and immunofluorescence.

**Results:**

FTY720 treatment results in a significantly better functional outcome persisting up to day 31 after PT. This is accompanied by a significant decrease in reactive astrogliosis and larger post-synaptic densities as well as changes in the expression of vascular endothelial growth factor α (VEGF α). Within the periinfarct cortex, S1P is significantly increased compared to healthy brain tissue.

**Conclusion:**

Besides its known neuroprotective effects in the acute phase of experimental stroke, the initiation of FTY720 treatment in the convalescence period has a positive impact on long-term functional outcome, probably mediated through reduced astrogliosis, a modulation in synaptic morphology and an increased expression of neurotrophic factors.

## Introduction

Stroke is the leading cause of serious long-term disability in developed countries. [1] Among stroke survivors, 50% suffer from a hemiparesis 6 months after stroke. [2] Many clinical trials of neuroprotective substances failed in the past. Therefore, stroke prevention and revascularization are still the main therapeutic options in stroke care. The failure of many experimentally successful neuroprotective agents in clinical trials may be due to the fact that many neuroprotectants inhibit not only mechanisms of damage, but also mechanisms of recovery. [3] FTY720 has emerged as a promising agent which has shown acute neuroprotective properties in different stroke models in mice and rats that have been reproduced by several independent laboratories. [4], [5], [6] However, whether FTY720 also has an effect on long-term outcome when administered in the remodeling phase starting several days post-stroke has not yet been studied.

Any damage to the brain leads to transcriptional, biochemical and morphological changes in astrocytes termed reactive astrogliosis. [7] The signaling cues leading to this damage are only partly known, but appear to be influenced by the cause of damage. [8] The resulting glial scar is widely considered to have a negative impact on mechanisms of recovery. [9] However, positive aspects of reactive astrocytes have also been shown. [10] S1P could be a direct mediator of reactive gliosis via activation of specific G protein-coupled S1P receptors, S1PR_1–5_. [11], [12] Some recent reports suggest that S1P and the S1P receptor agonist FTY720 influences glial scarring in experimental autoimmune encephalitis and spinal cord injury. [13], [14].

We examined whether behavioral recovery could be pharmacologically enhanced by delayed administration of FTY720 in a model of stroke assessing functional outcome over 31 days, astrocytic reactivity, synaptic morphology and as a possible mechanism of recovery, the influence of FTY720-treatment on the expression of neurotrophic factors. We furthermore studied the concentrations of S1P, FTY720 and phospho-FTY720 (pFTY720) and the expression levels of key enzymes of S1P metabolism in the periinfarct cortex.

## Methods

### Animals and Experimental Model of Photothrombotic Stroke

Male C57BL/6 mice (6–12 weeks old, strain J) were used in accordance with the National Institute of Health Guide for the Care and Use of Laboratory Animals (NIH Publications No. 80-23, revised 1996). All animal experiments were approved by the local government authorities (Regierungspraesidium Darmstadt). Stroke was induced by photothrombosis (PT) as described previously. [15] Briefly, after injection of buprenorphine, inhalative anesthesia using 2% isoflurane was performed. A cold light source (KL1500, LCD, Zeiss, Jena, Germany) was connected to a 40× objective, resulting in a 3 mm diameter light beam. The beam was stereotactically placed 1.5 mm lateral to the bregma. 5 minutes after the injection of 0.2 ml rose-bengal (Sigma-Aldrich, Taufkirchen, Germany; 10 mg/ml), the scull of the animal was illuminated for 15 minutes, inducing a focal stroke within the animal’s right-hemispheric motor cortex. At the indicated time points, animals were killed using an overdose of isoflurane.

### Sample Size Calculation, Experimental Groups and Randomization Procedure

Sample size calculations for the behavioral analysis as our main outcome measure were performed using a pilot group of 10 animals analyzed independently of the actual experiments. Defining an absolute difference of 10% as relevant for the cylinder task (CT) and 5% in the grid walking test (GWT) and expecting a standard deviation of 11% in the CT and 5% in the GWT derived from this pilot study, we calculated the minimal sample size to be *n* = 19 for the CT and *n* = 16 for the GWT to achieve a statistical power of 80% with a 0.05 probability of a type I error.

An overview of the experimental groups is given in Table 1.

**Table 1:** Experimental groups and number of mice entered into the study.

	n of sham-operated mice	n of control mice(saline = 0.9% NaCl)	n of FTY720-treated mice(1 mg/kg b.i.d. d3 to d7)
**Behavioral analysis**			
observation period 28 days following PT;assessment at day 7, day 14 and day 28	–	20	20
**Immunofluorescence**			
at day 8 after PT (GFAP, PSD95)	–	6	6
**Taqman-PCR** and **lipid tandem mass spectrometry**			
at day 4 after PT	d4 10	d4 10	d4 10

3 days after PT, mice were randomized using “pseudorandom” numbers (Urbaniak GC, Plous S. Research Randomizer, Version 3.0; 2011. www.randomizer.org; accessed April 22, 2011) and treatment with i.p. FTY720 (Selleck Chemicals) 1 mg/kg b.i.d. for 5 days versus 0.9% saline was started.

### Behavioural Analysis

Analysis of the behavioural outcome after PT was performed as described previously. [15] The video analysis was done by an examiner blinded to the treatment groups. The GWT was performed in a cage with an area of 600 cm^2^. The bottom was replaced by a mesh with an opening width of 1 cm^2^. The cage was placed at a height of 20 cm. A mirror placed under the cage allowed recording the mice walking on the grid on video for 5 min. The total number of steps was counted, whereas one step is defined as the movement of all four limbs. Furthermore the foot faults of the left paretic forepaw were counted. A foot fault is defined by a limb going through the grid or the paw resting on the grid only with its wrist.

For the CT, animals were placed in a plexiglas cylinder. While mice explored the surface by rearing up on their hindlimbs, the time of wall placement was recorded for the right forelimb, left forelimb and both forelimbs simultaneously. The difference between paretic (left) and non-paretic (right) plus bilateral placement was evaluated for each mouse.

### Transcardial Perfusion and Immunohistochemistry

For immunofluorescence, FTY720- and saline-treated mice (*n* = 6 per group) were sacrificed 7 days after PT. After perfusion with 0.1 M phosphate buffered saline (PBS), transcardial perfusion with cold 4% paraformaldehyde (PFA) in 0.1 M PBS was performed for 20 min, followed by 100 min of postfixation in 4% PFA. 40 µm slides were cut using a vibratome. For postsynaptic density protein 95 (PSD-95) immunofluorescence, a permeabilization step with 0.05% Triton X-100 was followed by preincubation with 10% normal horse serum and 4% Bovine Serum Albumin. Primary antibodies used here were a mouse anti-GFAP-Cy3 antibody (Sigma-Aldrich, clone G-A-5) and a rabbit anti-PSD-95 antibody (Abcam, ab18258), as a secondary antibody a donkey-anti-rabbit-Alexa488 antibody (Dianova, 711-486-152).

### Measurement of Reactive Astrogliosis

All slides of one experiment were incubated within the same dish, and microscopy performed strictly under the same conditions. We replicated our staining three times. Continuous images were taken from the entire ipsilateral cortex and arranged using the “panorama” function of the Axio Vision 4.8 software (Carl Zeiss, Jena, Germany). Using ImageJ (NIH, Bethesda, Maryland, USA), a 100 µm^2^ grid was projected on the entire image. The images 100–200 µm from the infarct border and 100–300 µm below the pia mater were taken for quantitative measurements with ImageJ. After setting the threshold within a replication at the same grey value, glial fibrillary acidic protein (GFAP)-immunoreactive area was measured using ImageJ.

### Measurement of PSD-density and Size

Postsynaptic density protein 95 (PSD95) immunofluorescence was performed as previously described. [16] After staining of brain sections, 16–20 z-stacks of 2 µm thickness of the periinfarct cortex (100–200 µm from the infarct border and 100–300 µm below the pia) were taken by a Zeiss confocal microscope, starting 5–10 µm below the surface of the slide. After deconvolution with the Richardson-Lucy Algorithm, unimodal thresholding was performed using matlab (The MathWorks, Natick, Massachusetts, USA). The Vamp2d plugin was used to visualize synapse size and the vamp3d dissection method to visualize synapse number. The ImageJ function “particle analyzer” was used to do the actual measurement.

### RT-PCR Analysis

After the indicated time points, mice brain tissue samples for PCR analysis were excised and immediately snap-frozen in liquid nitrogen. After homogenization for 1 min with 50 Hz using TissueLyser LT (Qiagen, Hilden, Germany), 1.2 µg of total RNA was isolated with TRIZOL™ reagent (Sigma-Aldrich, Steinheim, Germany) according to the manufacture’s protocol and used for reverse transcriptase-polymerase chain reaction (RT-PCR; Revert Aid™ first strand cDNA synthesis kit, Thermo Fisher Scientific, St. Leon-Rot. Germany) utilizing an oligo (dT) primer for amplification.

Real-time PCR (TaqMan®) was performed using Applied Biosystems 7500 Fast Real-Time PCR System. Probes, primers, and the reporter dyes 6-FAM and VIC were from Life Technologies (Darmstadt, Germany). The cycling conditions were as following: 95°C for 15 min (1 cycle), 95°C for 15 s and 60°C for 1 min (40 cycles). The threshold cycle (C_t_) was calculated by the instrument software (7500 Fast System SDS Software version 1.4). Analysis of the relative mRNA expression was performed using the ΔΔC_t_ method. The housekeeping gene GAPDH was used for normalization.

### S1P and FTY720 Quantification by Liquid Chromatography Tandem Mass Spectrometry (LC/MS/MS)

For quantification of S1P, FTY720 and its phosphate derivative FTY720-phosphate (pFTY720) about 10 mg tissue were homogenized with PBS and liquid-liquid extracted with methanol:chloroform:HCl (15:83:2). This analytical procedure has been slightly modified from the method published elsewhere. [17].

### Statistical Analysis

All results are displayed as means ± SD. Statistical significance was assessed with Student’s two-tailed unpaired t-test for two-group analyses and one-way ANOVA with Bonferroni correction for multigroup analyses. Differences with *P*<0.05 were considered to be significant.

## Results

### Delayed Treatment with FTY720 Improves Functional Neurological Outcome after PT over the Entire Observation Period of 31 Days

All deaths observed during our study occurred within the first 3 days after surgery, before randomization and we did not need to exclude animals from our study. Before the operation, mice of FTY720- and saline-treated groups show the same behavioral status with an almost zero dexterity result in the CT as well as the GWT.

At day 7, both tests reveal the extent of the motor cortex lesion, with all animals showing a stronger use of their ipsilateral limb in the CT and performing clearly more foot faults in the GWT as compared to baseline.

**Figure 1:**
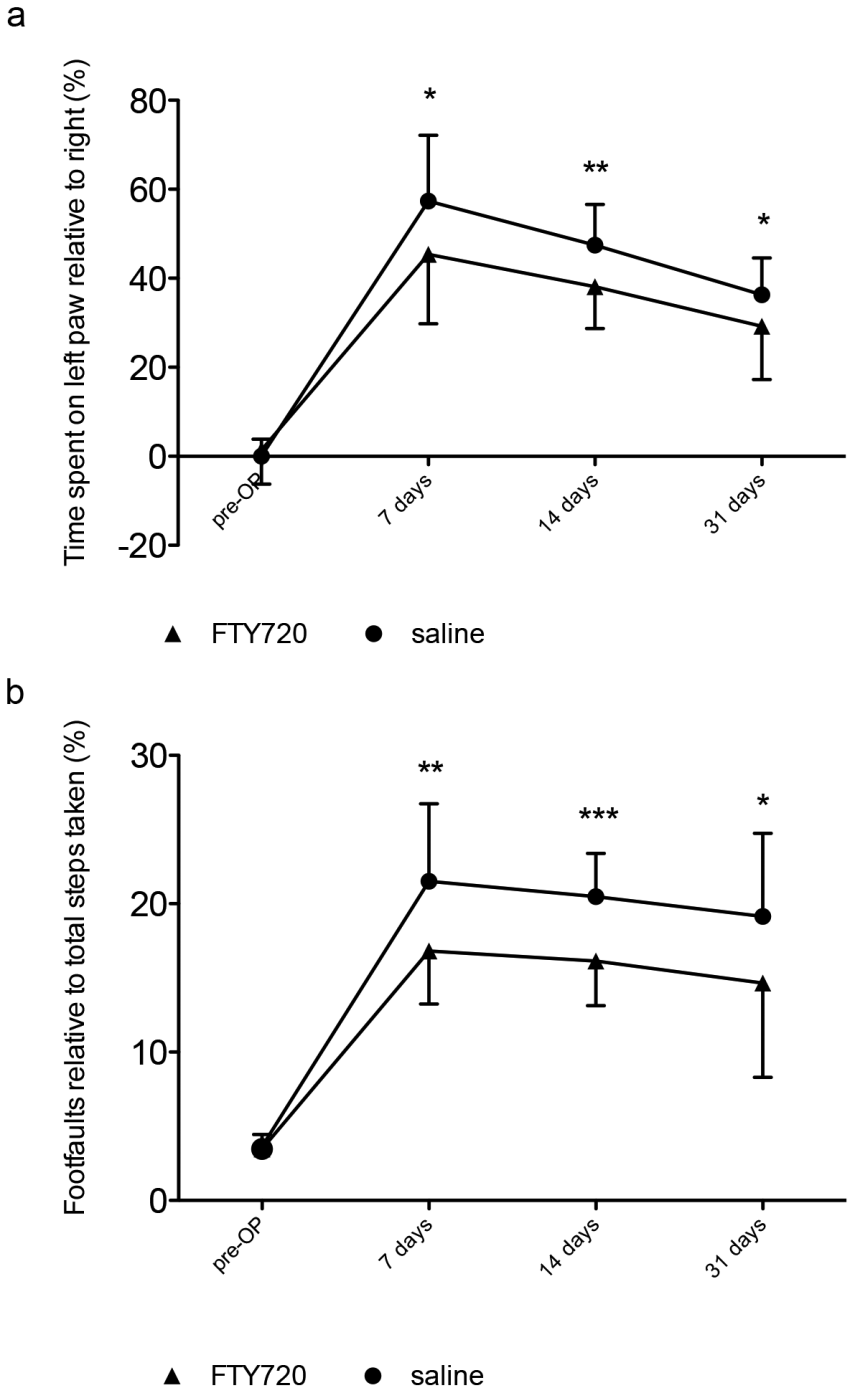
Improved recovery after stroke with late-initiated FTY720-treatment. a) Forelimb asymmetry was assessed using the cylinder task. b) Foot faults were measured using the grid-walking test. Data are presented as means ± S.D.; * *P*<0.05; ** *P*<0.01; *** *P*<0.001; pre-OP - before photothrombotic stroke. Differences between treatment groups at each time point of neurological assessment were analyzed using Student’s two-tailed unpaired t-test; *n* = 20/group.

Animals treated with FTY720 show a significantly lower deficit in both tests at day 7. In the CT (Fig. 1a), FTY720-treated mice show a significantly lower preference of their non-paretic forepaw as compared to saline-treated mice (FTY720-treated mice: 45.3±15.2%, saline-treated mice: 57.5±14.2%, *P* = 0.0125, *n* = 20/group). In the GWT (Fig. 1b), FTY720-treated mice show significantly lower percentage of foot faults of the paretic forepaw as compared to saline-treated mice (FTY720-treated mice: 16.8±3.5%, saline-treated mice: 21.3±5.4%, *P* = 0.0034, *n* = 20). This effect persists up to day 31, as reflected in the CT (29.3±11.7% versus 37.1±7.8%, *P* = 0.0177, *n* = 20) as well as in the GWT (14.8±6.2% versus 19.1±5.7%, *P* = 0.028, *n* = 20).

### FTY720 Reduces Reactive Astrogliosis in Experimental Stroke

**Figure 2:**
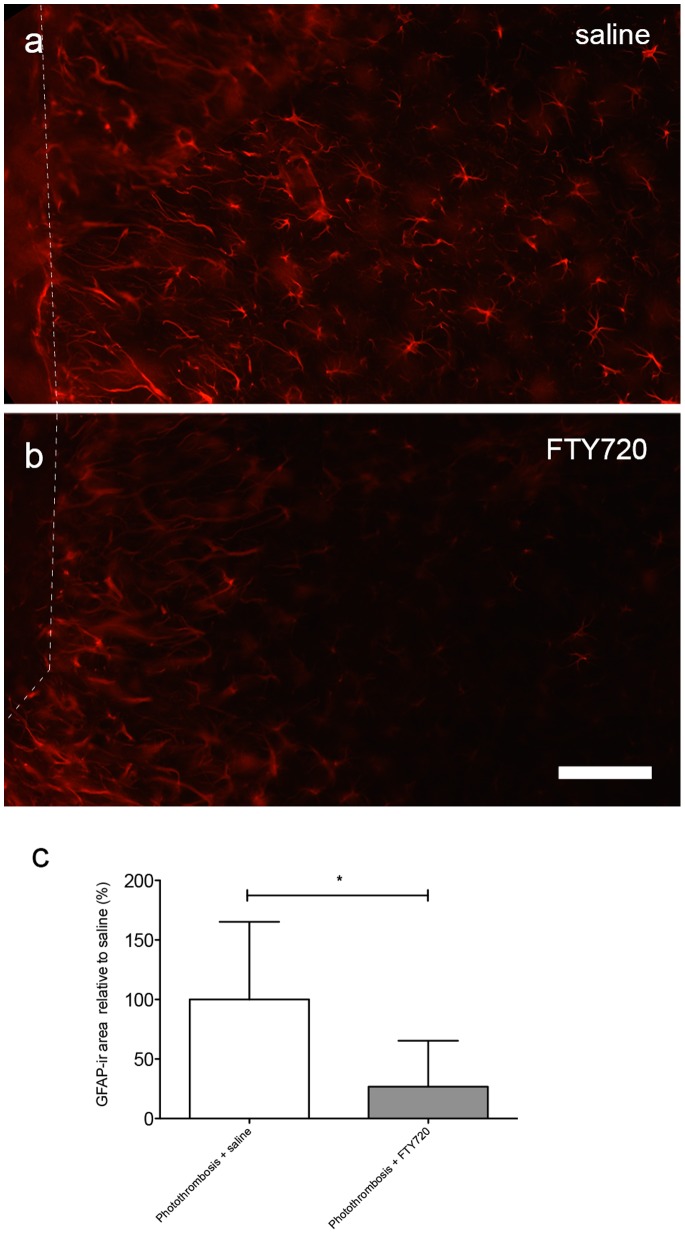
FTY720 reduces reactive astrogliosis in the periinfarct cortex. a) GFAP-ir signal in the periinfarct cortex (100–200 µm from infarct border, denoted by broken line) in saline-treated mice. b) GFAP-ir of reactive astrocytes captured under identical viewing parameters (objective, exposure time, threshold) in the periinfarct cortex of mice treated for 5 days with FTY720, 1 mg/kg, beginning at day 3. Scale bar = 100 µm. c) Comparison of the GFAP-ir area in the periinfarct cortex of FTY720 and saline treated mice. * *P*<0.05. Differences between treatment groups were analysed using the Student’s unpaired two-tailed t-test; *n* = 6/group.

We quantified the GFAP immunoreactivity (−ir) within layer 2/3, at a distance of 100–200 µm from the infarct border, which is considered to be the main area of axonal sprouting and where synaptic recovery takes place. [18] Astrocytic reactivity is induced strongly starting between day 2–3 after PT (data not shown) within the ipsilateral and the contralateral cortex with a maximal GFAP-ir at the direct infarct border (Fig. 2a, b). FTY720 treatment significantly reduces reactive astrogliosis, as measured by the GFAP-ir area in the indicated periinfarct zone (FTY720-treated mice: 26.7±38.6%, saline-treated mice: 100±65.24%, *P* = 0.0395, *n* = 6; Fig. 2c).

### Synapse Size is Increased in FTY720-treated Mice

**Figure 3:**
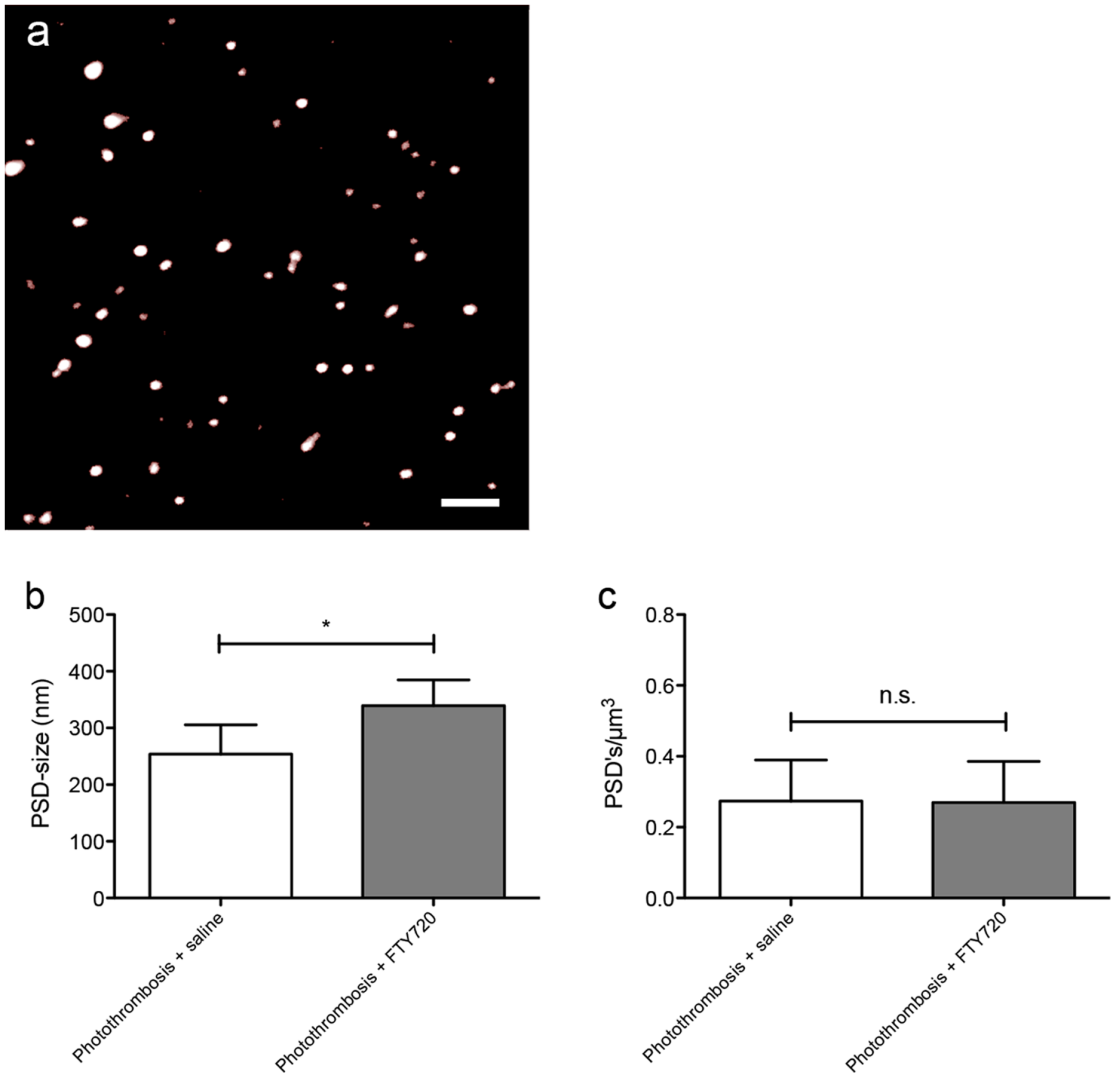
FTY720-treatment results in larger PSD’s in layer ½ of the periinfarct cortex. a) Exemplary image of the periinfarct cortex in an FTY720-treated animal, acquired by confocal microscopy and the vamp2d-algorithm [16]. Scale bar = 2 µm. b) Quantification of the PSD-size. c) Quantification of PSD-numbers by the vamp3d-algorithm. Data are presented as means+S.D. * *P*≤0.05; n.s. - non-significant. Differences between treatment groups were analyzed using Student’s two-tailed unpaired t-test; *n* = 6/group.

As an indirect measurement of synaptic morphology within the periinfarct cortex, the morphology of postsynaptic densities at day 7 was analyzed using the vamping method (Fig. 3a). Within the selected area, quantified postsynaptic densities are significantly larger in FTY720-treated animals (338.1±47.6 nm) as compared to the saline-treated animals (257.7±47.6 nm, *P* = 0.0152, *n* = 6; Fig. 3b). The number of postsynaptic densities does not differ between both treatment groups (FTY720-treated animals: 0.2650±0.09035 PSD’s/µm^3^, saline-treated animals: 0.2768±0.9979 PSD’s/µm^3^, *P* = 0.8838, *n* = 6; Fig. 3c).

### FTY720 Treatment Increases the Expression of VEGFα

**Figure 4:**
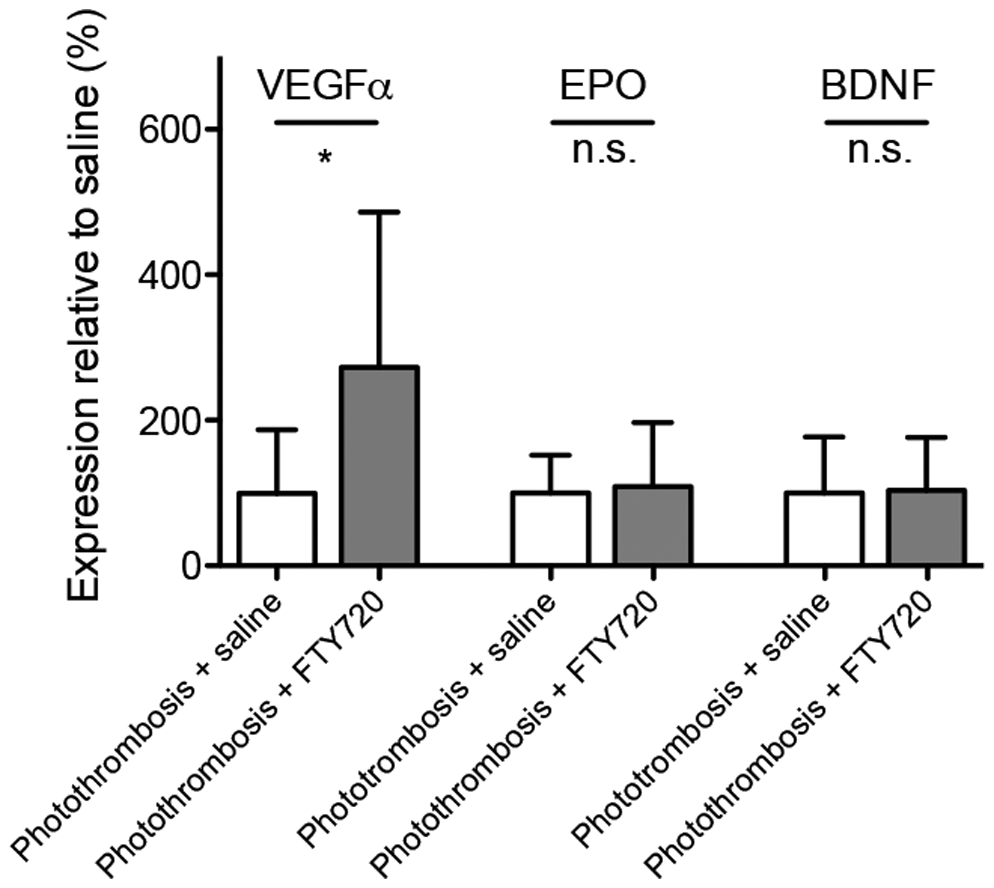
FTY720-treatment leads to an increase of VEGFα-mRNA, but not BDNF- and EPO-mRNA in the periinfarct cortex. RT-PCR analysis of neurotrophic factors in periinfarct tissue of FTY720-treated mice compared to samples of saline-treated mice. Differences between treatment groups were analyzed using Student’s unpaired two-tailed t-test. Data are presented as means+S.D. *, *P*≤0.05; n.s., non-significant; *n* = 10/group.

RT-PCR of the periinfarct tissue was performed in order to investigate changes in the expression levels of main neurotrophic factors. FTY720 significantly increases VEGFα-expression at day 4 after PT (Fig. 4). Whereas VEGFα-expression in the periinfarct cortex is not significantly increased by PT itself (data not shown), it is significantly higher in FTY720-treated mice (274.1±218.5%) as compared to saline-treated mice (100±85.2%, *P* = 0.0305, *n = 10)*. Tissue mRNA levels of erythropoietin (EPO, 108.5±88.5% of saline-treated mice, *P* = 0.8174, *n = 10*) or brain-derived neurotrophic factor (BDNF, 103±72.64% of saline-treated mice, *P* = 0.9237, *n = 10*), two other important mediators of CNS recovery within the periinfarct cortex do not reveal any changes in the mRNA expression-levels by FTY720-treatment (Fig. 4).

### S1P Levels are Increased in the Periinfarct Cortex after PT

**Figure 5:**
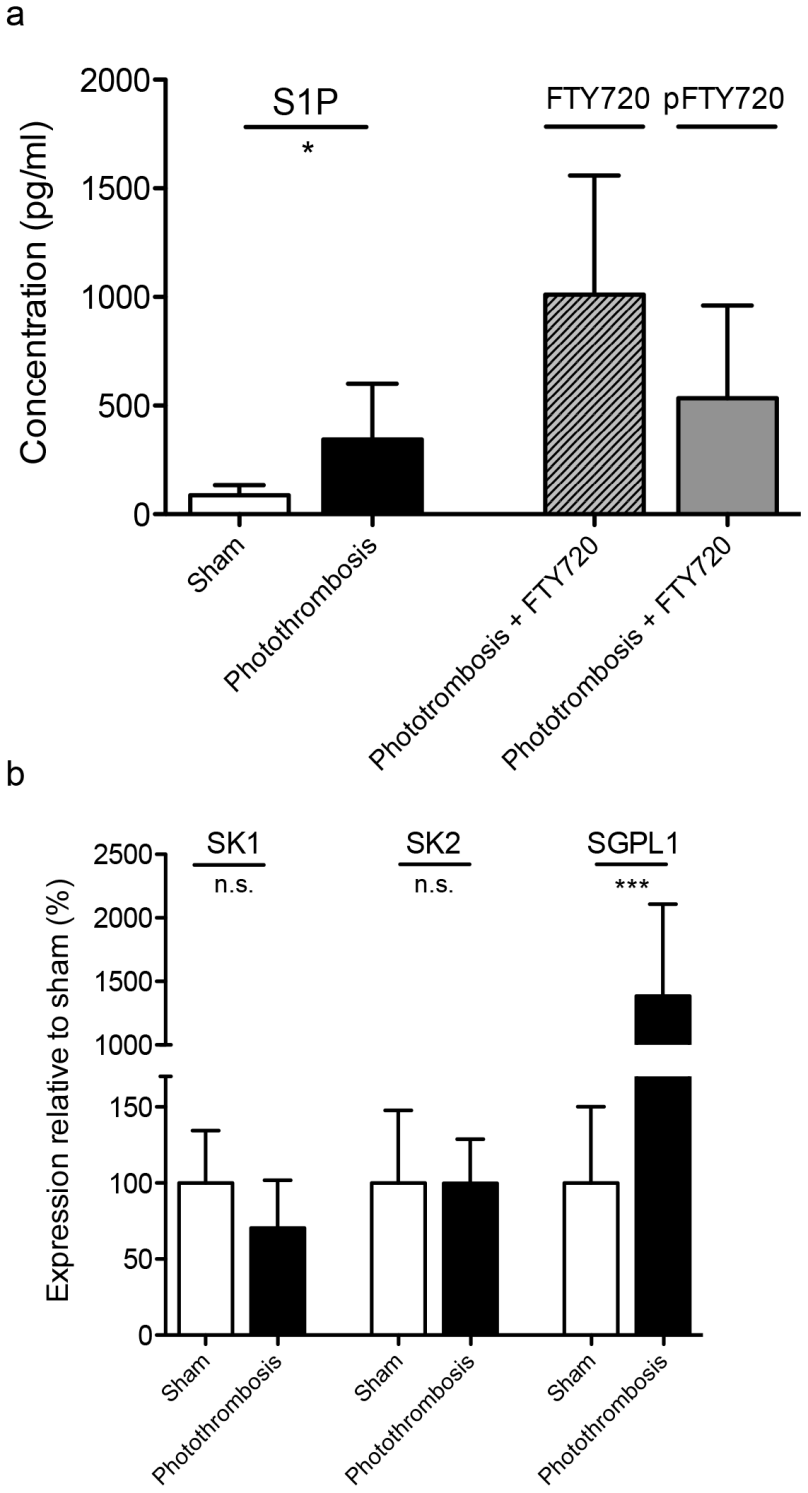
S1P is increased in the periinfarct cortex, not explained by changes in the expression of enzymes of S1P metabolism. a) S1P, FTY720 and pFTY720 were measured at day 4 by tandem mass spectrometry. b) SK1, SK2 and SGPL1 were measured at day 4 by RT-PCR. Differences between treatment groups were analyzed using Student’s unpaired two-tailed t-test. Data are presented as means ± S.D. **, *P*≤0.01; *, *P*≤0.05; n.s., non-significant; *n* = 10/group.

In parallel to the therapeutic approach with the S1P analog FTY720, we investigated changes in concentrations of the natural signaling molecule S1P within the periinfarct cortex. S1P is significantly increased at day 4 after PT (343.1±275 pg/ml) in saline-treated animals compared to sham-operated animals (90.1±41 pg/ml, *P* = 0.01, *n* = 10; Fig. 5a). In order to monitor the pharmacokinetics of FTY720 within the periinfarct cortex in FTY720-treated animals, we performed tandem mass-spectrometry for FTY720 and pFTY720 one day after the initiation of the treatment (Fig. 5a). We found that one day of treatment with 1 mg/kg FTY720 b.i.d. leads to a concentration of 1010±549.6 pg/ml of FTY720. However, the active metabolite, pFTY720 had a concentration of 534±427.6 pg/ml (*n* = 10). As expected, saline treated animals did not show any FTY720 or pFTY720 in the periinfarct cortex (data not shown).

### Enzymes of S1P Metabolism are only Partly Regulated after Photothrombotic Stroke

RT-PCR of the periinfarct tissue was performed to find changes in the expression levels of key enzymes of S1P metabolism, sphingosine kinase 1 (SK1), sphingosine kinase 2 (SK2) and the S1P-lyase (SGPL1, Fig. 5b). Photothrombotic stroke does not induce any changes of SK1 (70.5±31.5% of sham-treated mice, *P* = 0.0598, *n* = 10) and SK2 (99.7+96.7% of sham-treated mice, *P* = 0.9962, *n* = 10) expression. However, SGPL1-mRNA is significantly increased in the periinfarct cortex (1366.7±750% of sham-treated mice, *P* = <0.0001, *n* = 10).

## Discussion

We show that a short course of treatment with the synthetic S1P-analog FTY720 from day 3 to day 7 after experimental stroke enhances functional neurological recovery. This proregenerative effect of FTY720 is stable over an observation period of 31 days and accompanied by a reduction of reactive astrogliosis and an increase in synapse size. As a possible mediator of this effect we show that FTY720-treatment leads to an increase in the expression of VEGFα. Furthermore, we demonstrate by means of lipid tandem mass spectrometry that the tissue S1P concentration rises in the periinfarct cortex after stroke.

FTY720 treatment improved functional outcome in both the GWT as well as the CT. Both tests have been widely used to evaluate long-term neurological deficit in different stroke models. [19] While the results of the CT reflect an improved motor recovery in the FTY720 treatment group, the GWT shows that coordination and sensory function are also improved.

While FTY720 has shown neuroprotective properties in the acute phase of stroke in several stroke models and animal species, [4], [5], [6] this is the first report of enhanced functional recovery under FTY720-treatment. The relatively late initiation of FTY720-treatment at day 3 as well as the PT stroke model were chosen in order to clearly separate a proregenerative effect from the known acute neuroprotective effect of FTY720. At day 3 after stroke, further ongoing cell death and increase of infarct size is not likely. [20], [21] Therefore, day 3 is frequently used as a time-point to initiate neuroregenerative therapies in experimental studies. Additionally, although PT has been shown to be responsive to neuroprotectants, [22], [23] it is much less responsive than other stroke models [24], [25]. We therefore conclude, that FTY720 might be one of the very few promising neuroregenerative agents characterized so far.

As the effect of FTY720 appears to happen prior to day 7, we anticipated correlative morphological effects at this time point. We observed that FTY720 also leads to a decrease of reactive astrogliosis in the periinfarct tissue. The extent of reactive astrogliosis has been shown to negatively correlate with functional recovery in various models of neurological disease. [7] Importantly, FTY720 has been shown to inhibit reactive astrogliosis in models of multiple sclerosis [13], [26] and spinal cord injury [14]. Whether this effect is the result of a direct action of FTY720 at the astrocyte via S1P-receptors or an indirect mechanism e.g. via reduced T-cell influx and consecutively reduced cytokine expression has to be shown in future experiments. The local immune response seemed not to be influenced by FTY720, as we did not observe a decreased activation of micoglia/macrophages in the periinfarct cortex (fig. S1).

Morphological changes in postsynaptic structures are believed to play a fundamental role in physiological and regenerative processes. [27], [28] PSD size, spine size and the location of AMPA receptors at the postsynaptic membrane are closely linked and correlated to synaptic strength. [29], [30] The approach used here has been shown to be a reliable screen for changes in synapse size and number. [16] Our observation that FTY720 treatment leads to significant larger PSDs within the area where recovery is mediated [18] might be an explanation for the improved behavioral outcome. However, we could not show an increased PSD-density as a probable consequence of an increased axonal sprouting into the periinfarct cortex.

How could the effect of FTY720 on behavioural and morphological outcome after photothrombosis be mediated? As one possible mechanism, we analyzed the mRNA-levels of well-known neurotrophic factors. Intriguingly, VEGFα mRNA in the periinfarct cortex is significantly increased by FTY720-treatment. VEGFα has been shown to enhance synaptic plasticity [31] and to be beneficial in experimental stroke. [32] Interestingly, VEGFα has been recently demonstrated to be mainly located within astrocytes. [33] A pro-angiogenic state has been shown to be beneficial in stroke patients [34] and, importantly, a late treatment with VEGFα leads to improved outcome in experimental stroke. [35] Until now it is a matter of speculation, whether both increased VEGFα expression and reduced glial reactivity are results of the same intracellular pathway, modulated by FTY720. VEGFα expression has been show to be influenced by S1P signaling in other settings. [36], [37] We did not investigate whether periinfarct angiogenesis is affected by FY720-treatment and increased VEGFα expression. Interestingly, it should be noted that FTY720 is discussed as an anti-angiogenic factor. [38] Some pro-regenerative treatments in experimental stroke have been shown to be mediated by BDNF [15], and FTY720 was reported to quickly induce BDNF expression compared to vehicle in a model of Rett-disease. [39] In contrast, our results argue against a major impact of a modulated BDNF-expression on the proregenerative effect of FTY720 treatment after PT.

Our results from lipid tandem mass spectrometry show a significant elevation of cortical periinfarct tissue S1P levels after PT. This is at least in part in line with a previous report, in which the authors show an increase of S1P in the whole brain at day 14 after experimental stroke, but not at day 7. [40] To our knowledge, this is the first time that S1P concentrations were evaluated selectively in the periinfarct cortex with mass spectrometry, the current gold standard method to assess sphingolipid concentrations. Unfortunately, we were not able to determine the source of elevated S1P. However, we do not see any hints that the elevation is a result of a regulated expression of S1P-generating as well as degenerating enzymes. Only the SGPL1 appears to be upregulated in the periinfarct tissue, probably as a compensatory mechanism in order to downregulate the high S1P elevation. These results do not point to a change in local synthesis of S1P. As rather high concentrations of S1P can be found intracellularly and blood plasma, [41] our results might therefore be interpreted as if the source of increased S1P are either death cells of the infarct core or the blood-brain barrier damage induced by photothrombosis.

The i.p. injections of FTY720 lead to stable concentrations of both FTY720 as well as pFTY720 in the periinfarct cortex, showing an effective activation of FTY720 by sphingosine kinase 2-mediated phosphorylation. [42] pFTY720 has been shown to be a functional antagonist of S1PR_1_ and S1PR_3_ and both receptors have been discussed to be mediators of astroglial reactivity. [13], [43] It is therefore tempting to speculate that pFTY720 antagonizes the gliotrophic effect of the elevated S1P concentrations in the periinfarct tissue.

One has to keep in mind the limitations of the PT model. Major differences to human stroke are a. the occlusion of all kind of vessels within the illuminated area, b. the lack of a penumbra and c. the induction of a vasogenic edema (for review see [44]). As all these differences particularly affect mechanisms of the development of CNS damage, we claim that the model is suitable for the selective examination of stroke recovery. Unfortunately, transient proximal middle cerebral artery occlusion for 1 hour was not a suitable model to confirm our results due to a low seven day survival and a low deficit of the surviving animals (fig. S2 & S3).

In conclusion, we found that S1P is increased in the periinfarct cortex after PT with a pronounced motor deficit and that manipulation of the S1P pathway by FTY720 after the critical time window for neuroprotection improves long-term outcome after experimental stroke. In parallel, we observed a reduction of reactive astrogliosis, alterations in synaptic plasticity and differential expression of the neurotrophic factor VEGFα. We suggest that FTY720 is a promising candidate for neuroregenerative therapies after stroke.

## Supporting Information

Figure S1 **No difference between treatment groups in the number of CD11b-ir cells in the periinfarct cortex after photothrombosis.** Results of the immunofluorescence analysis, quantified by a rater blinded to treatment groups. Saline-treated mice: 0.44+0.1 CD11b-ir cells/µm^2^; FTY720-treated mice: 0.35+0.12 CD11b-ir cells/µm^2^, *P* = 0.204. Differences between treatment groups were analyzed using Student’s two-tailed unpaired t-test; *n* = 6/group.
(TIF)

Figure S2 **7-day survival of mice with 1 h tMCAO.** 30% of the operated animals died until day 3, before the start of randomization and treatment. The red line represents the beginning of treatment. For comparison, percent survival of the FTY720-group (dashed line) and saline-group (dotted line) reset to 100%.
(TIF)

Figure S3 **No difference of functional deficit between both treatment groups.** a) Results of the cylinder task at day 7 after tMCAO. Saline-treated mice: 24.6+16.8%; FTY-treated mice: 25.5+12.9%, P = 0.925. b) Results of the grid-walking test. Saline-treated mice: 0.92+1.4%; FTY-treated mice: 1.43+2.6%, *P = *0.686. Differences between treatment groups were analyzed using Student’s two-tailed unpaired t-test; *n* = 5/group.
(TIF)

Methods S1 **Supplemental methods.**
(DOCX)
