# Supplementary figures and images for: FTY720 Treatment in the Convalescence Period Improves Functional Recovery and Reduces Reactive Astrogliosis in Photothrombotic Stroke
Source: PLoS One. 2013 Jul 31;8(7):e70124. doi: 10.1371/journal.pone.0070124 (PMC3729514; doi:10.1371/journal.pone.0070124)

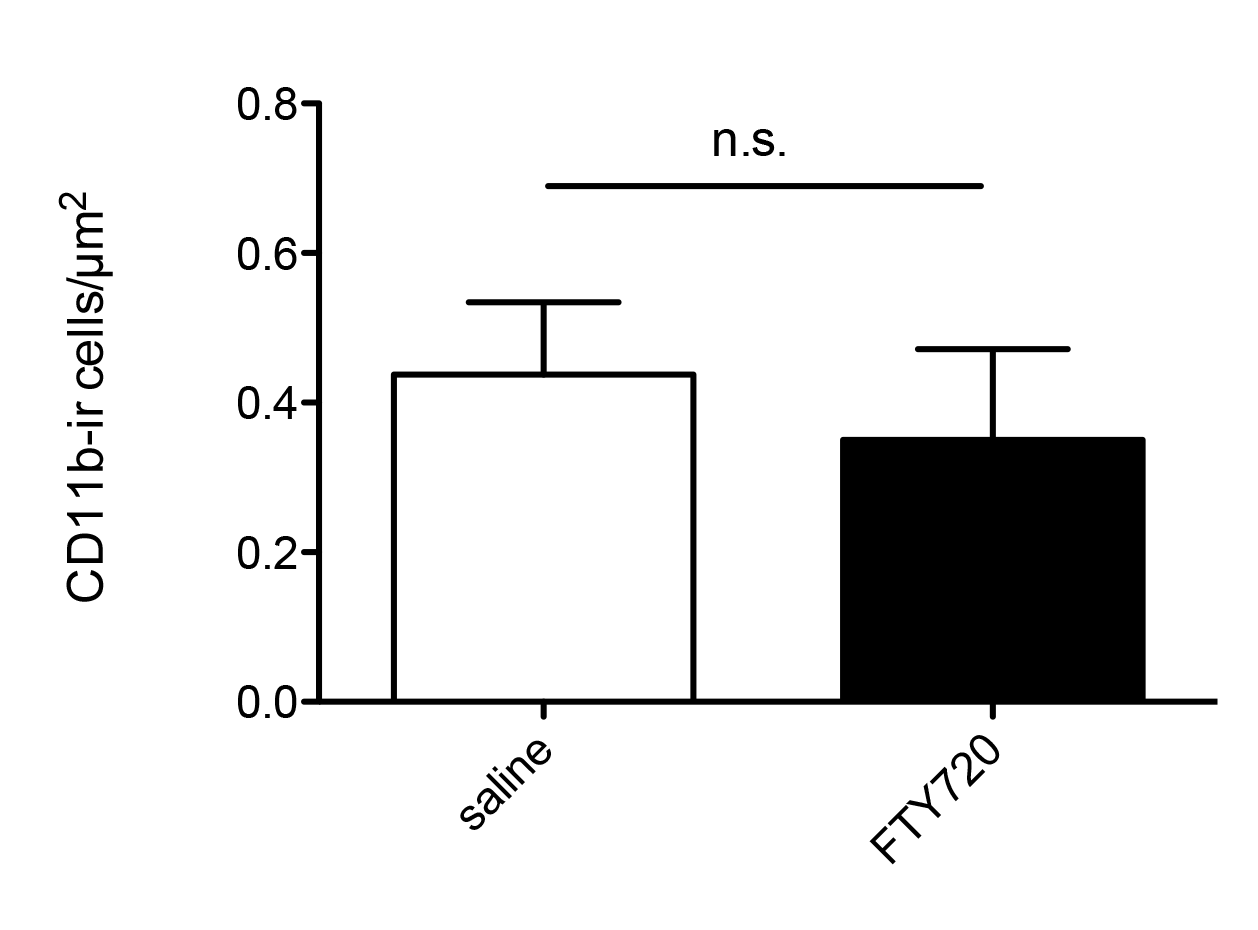

Supplement: Figure S1 — No difference between treatment groups in the number of CD11b-ir cells in the periinfarct cortex after photothrombosis. Results of the immunofluorescence analysis, quantified by a rater blinded to treatment groups. Saline-treated mice: 0.44+0.1 CD11b-ir cells/µm2; FTY720-treated mice: 0.35+0.12 CD11b-ir cells/µm2, P = 0.204. Differences between treatment groups were analyzed using Student’s two-tailed unpaired t-test; n = 6/group. (TIF) [file pone.0070124.s001.tif]

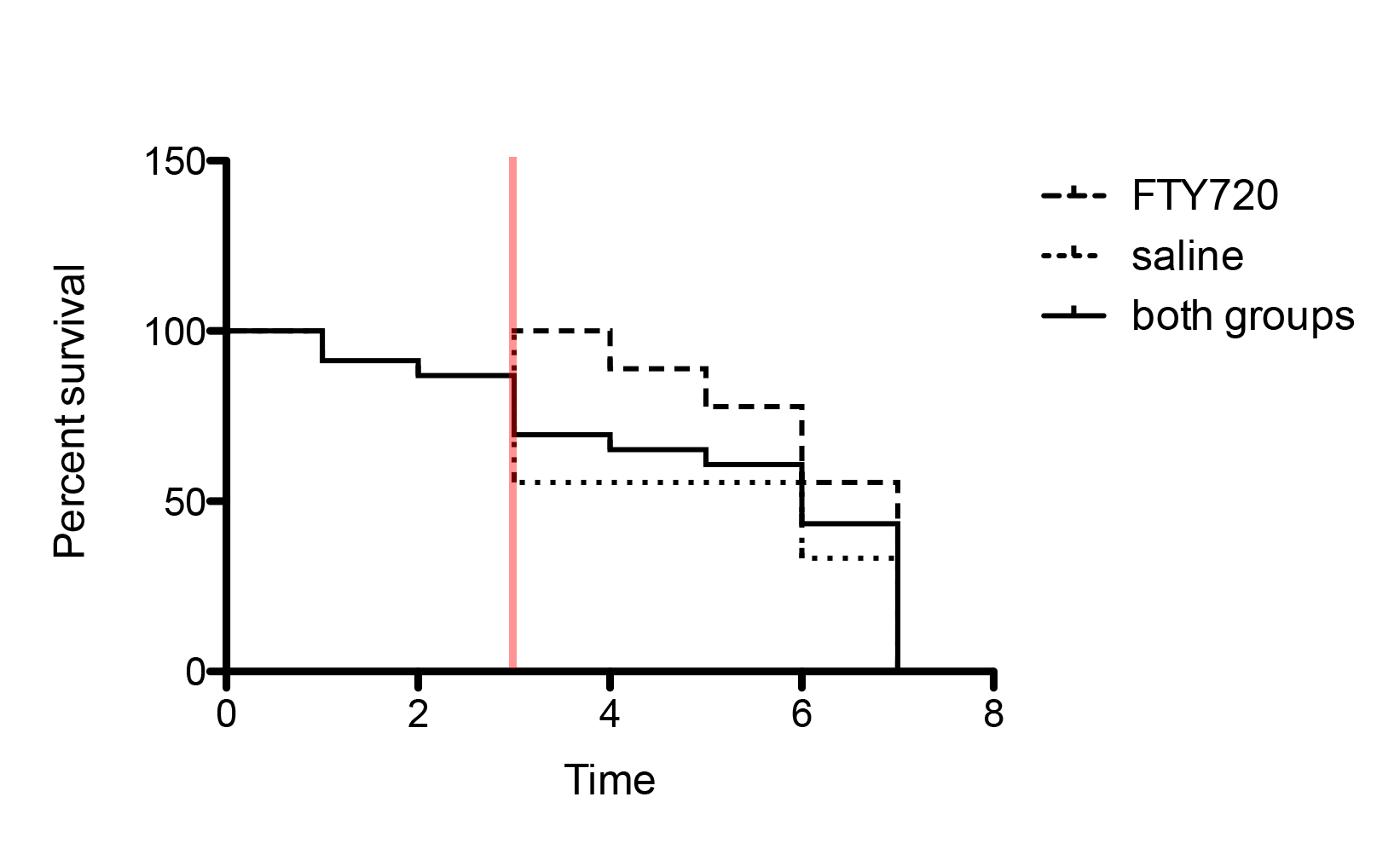

Supplement: Figure S2 — 7-day survival of mice with 1 h tMCAO. 30% of the operated animals died until day 3, before the start of randomization and treatment. The red line represents the beginning of treatment. For comparison, percent survival of the FTY720-group (dashed line) and saline-group (dotted line) reset to 100%. (TIF) [file pone.0070124.s002.tif]

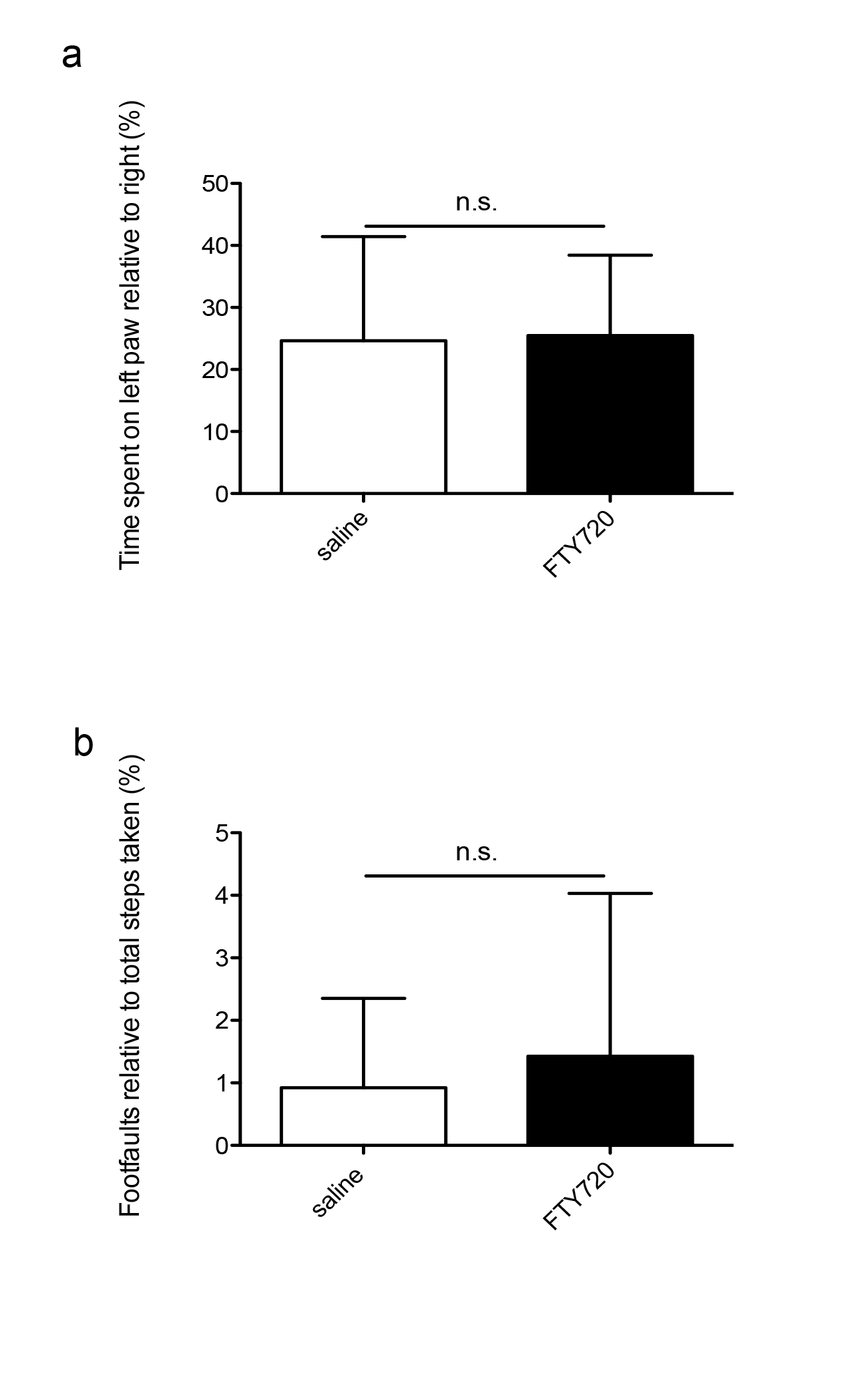

Supplement: Figure S3 — No difference of functional deficit between both treatment groups. a) Results of the cylinder task at day 7 after tMCAO. Saline-treated mice: 24.6+16.8%; FTY-treated mice: 25.5+12.9%, P = 0.925. b) Results of the grid-walking test. Saline-treated mice: 0.92+1.4%; FTY-treated mice: 1.43+2.6%, P = 0.686. Differences between treatment groups were analyzed using Student’s two-tailed unpaired t-test; n = 5/group. (TIF) [file pone.0070124.s003.tif]
