# Supplementary material for: FTY720 Treatment in the Convalescence Period Improves Functional Recovery and Reduces Reactive Astrogliosis in Photothrombotic Stroke
Source: PLoS One. 2013 Jul 31;8(7):e70124. doi: 10.1371/journal.pone.0070124 (PMC3729514; doi:10.1371/journal.pone.0070124)
Supplement: Methods S1 — Supplemental methods. (DOCX) [file pone.0070124.s004.docx]

Supplemental Methods

Quantification of CD11b-ir cells

Continuous images were taken from the entire ipsilateral cortex of animals with a photothrombotic stroke and arranged using the “panorama” function of the Axio Vision 4.8 software (Carl Zeiss, Jena, Germany). Using ImageJ (NIH, Bethesda, Maryland, USA), a 100 µm^2^ grid was projected on the entire image. The images 100–200 µm from the infarct border and 100–300 µm below the pia mater were taken for quantitative measurements with ImageJ. CD11b-ir cells were counted using the “cell counter” plug-in. The analysis was done by an examiner blinded for treatment groups.

Transient proximal middle cerebral artery occlusion

1 h transient MCAO was performed as described previously in 20 mice.[[1](#_ENREF_1)] Mice were anaesthetized with 1.5% isoflurane (Forene; Abbott, Wiesbaden, Germany) and 0.1 mg/kg buprenorphine (Temgesic; Essex Pharma, Munich, Germany) under spontaneous respiration. Focal cerebral ischemia was induced by inserting standardized monofilaments with a tip diameter of 0.23 mm into the right MCA, (Doccol, Redlands, CA, USA). After three days, mice were randomized to receive either FTY720 (1 mg/kg) or saline b.i.d. We observed survival and assessed functional neurological outcome at day 7 with the CT and the GWT.

1. Czech B, Pfeilschifter W, Mazaheri-Omrani N, Strobel MA, Kahles T, et al. (2009) The immunomodulatory sphingosine 1-phosphate analog FTY720 reduces lesion size and improves neurological outcome in a mouse model of cerebral ischemia. Biochemical and Biophysical Research Communications 389: 251-256.
